# French Operations, with Reflections

**Published:** 1831-04-01

**Authors:** 


### XVIII.

### HOPITAL DE LA CHARITE.

#### French Operations.

No idea can be more erroneous, no resolution more absurd, than to send young men to Paris to *learn* their profession. If they stop for a short time they cannot derive benefit; if for a longer time, they do not. We have seen a good deal of the effects of this method of study, as it is called; we have seen them before starting, and after coming back. In not one instance have we witnessed any thing like a rational improvement. These persons become Frenchified Englishmen; they learn to smoke and to drink coffee, it is true; if more apt and quick witted, to fence and to gamble; but to practise their profession with credit or advantage, they seldom or never do learn in Paris. Now, if any thing could be acquired, one would think it were anatomy; yet even in that they are rarely accomplished. From the impossibility of making anatomical preparations in Paris, our English students fall into a habit of dancing a gallopade through a body, and too often are rendered slovenly cutters up, rather than dexterous and neat dissectors. But we will allow that a diligent young man becomes a good anatomist in Paris. Can he also become a good surgeon? We maintain that he cannot. The impressions of his youth, the recollection of what he has seen in England must rise before his mind when he witnesses a totally different method in France. He grows distracted and confused between opposite modes of practice and conflicting opinions; he knows not whether Hunter and Sir Astley Cooper or Dessault and M. Dupuytren are right, and he returns to his native land to undergo the same process of doubt and difficulty when he sees again the usages of his countrymen. Some persons may regard this as a caricature, but it really is none. Let a young physician or surgeon complete his *education* here before he goes abroad to hear other precepts and see other practice. He will then derive advantage from the scenes that are presented to him; he will observe, compare, and reason; he will find himself qualified to discriminate the points in which foreigners excel us, and those in which we excel foreigners; he will be informed not confounded; his opinions will be matured and strengthened by comparison, not disordered and overthrown by contradiction.

These reflections were excited by the perusal of a clinical report from La Charité, in the Gazette Médicale de Paris. All sensible and well-informed English surgeons have remarked the great mortality after operations in the Parisian hospitals; they have observed with regret that the exclamation of Frère Jacques, 'l'opération est achevée;—Dieu vous garde!' might not inappropriately be attributed to any of the surgeons of Paris at this moment. Our professional cosmopolites may be shocked at this assertion, yet it seems to be acknowledged by many of the French themselves. In the prospectus of a French medical journal we find the following passage.

"La chirurgie Française parait avoir placé le but de tout perfectionnement dans la partie du diagnostic et de l'operation; nulle enguette des indications, nul souci de la therapeutique générale et consécutive; il semble enfin q'on n'ai à agir que sur un corps inerte."

When a journal in France starts into existence with such a declaration, when such is the device upon its banner, what must we think of the state of French surgery, considered as a means of alleviating human misery. If operations, bloody cruel operations, are looked on with admiration, it can only be by those who regard plague, pestilence, and famine as benefits also. No! operations are our opprobrium, our disgrace, not out proper and legitimate boast; the mere operator is little better than a human butcher by rule. There is too great a taste for the knife at present; —shame to those who encourage it;—woe to those who practise it. Unnecessary operations bring discredit on surgery; unsuccessful ones, even when perfectly justifiable, inspire doubt and repugnance to it in the public mind. Patients who see or hear of their relatives or friends submitting to barbarous and frightful maimings, only that they might live for a month or a week, feel no great relish for the knife, when its use might bring safety and a cure. What a sarcastic, what a damning catalogue of *great* operations performed within these last few years, might a diligent cynic compile for the gratification of the world! But we have done, and we trust that these observations may not be without their effect.

*Case* 1. In the beginning of December, 1829, a man was brought into the wards of M. Roux, with fracture of the lower extremity of the fibula, rupture of the ligaments, and compound dislocation of the ankle-joint. The foot was drawn considerably backwards, the lower end of the tibia projected through the wound. The accident had happened from the patient's having slipped suddenly into a deep rut. The parts were covered with a poultice and a bandage was applied. On the third day a portion of the skin had sloughed, without much preceding inflammation, and M. Roux prognosticated the most unfavourable consequences. On the succeeding day the inflammation of the limb was more severe; fever and delirium supervened; and on the fifth day the patient died. It should be remarked, that on the second day distinct emphysema had been observed at the upper part of the leg and inferior part of the thigh. This was attributed by M. Roux to the introduction of air into the subcutaneous cellular tissue through the medium of the wound.

The reporter severely criticises M. Roux for his inaction in the preceding case. We must say that the treatment appears to us to have been inefficient and unscientific. If the injury was *very* severe in the first instance, the limb should have been amputated; or if circumstances were tolerably favourable, which they seem to have been, amputation might have been performed when the gangrene commenced. The application of poultices to the compound dislocation in the first instance, was wrong. We have used this case as the peg on which to hang the few observations that we have made on French operations and French education, observations that are not uncalled for or impertinent at the present time.

